# Supplementary material for: Evidence for a reactionary account of retrieval state initiation
Source: Imaging Neurosci (Camb). 2025 Nov 21;3:IMAG.a.1023. doi: 10.1162/IMAG.a.1023 (PMC12641140; doi:10.1162/IMAG.a.1023)
Supplement: Supplementary Material [file IMAG.a.1023_supp.pdf]

Supplemental Material: Evidence for a reactionary account of retrieval state initiation.

Subin Han, Nicole M. Long

Here all mnemonic-state based analyses reported in the main manuscript were recalculated utilizing the full training (N=103) set of participants from the mnemonic state task. We find a stimulus-interval effect of instruction on mnemonic state evidence with no instruction-interval effects or interactions with SOA.

We find significant instruction by time interactions during the stimulus interval, with no effects of SOA on retrieval evidence. We likewise fail to find any effect of instruction on instruction-interval retrieval evidence. We find a main effect of SOA on instruction-interval retrieval evidence.

1. We conducted a 2 $\times$ 20 rmANOVA with factors of instruction (encode, retrieve) and time interval (twenty 100 ms time intervals across the 2000 ms stimulus interval) and retrieval state evidence as the dependent variable. We found a significant main effect of instruction ($F_{1,38}$ = 17.07, $p = 0.0002$, ${\eta_p}^2$ = 0.31) driven by greater retrieval evidence for retrieve (*M* = 0.0225, *SD* = 0.0405) compared to encode trials (*M* = -0.0124, *SD* = 0.0326). We found a significant main effect of time interval ($F_{19,722}$ = 17.72, $p <$ 0.0001, ${\eta_p}^2$ = 0.318). We found a significant interaction between instruction and time interval ($F_{19,722}$ = 2.540, $p = 0.0003$, ${\eta_p}^2$ = 0.0626) driven by greater retrieval evidence for retrieve compared to encode trials later in the stimulus interval.

2. We conducted a 2 $\times$ 4 $\times$ 20 rmANOVA with factors of instruction (encode, retrieve), SOA (500 ms, 1000 ms, 1500 ms, 2000 ms), and time interval (twenty 100 ms time intervals) and retrieval state evidence as the dependent variable. We found a significant main effect of instruction ($F_{1,38}$ = 18.03, $p = 0.0001$, ${\eta_p}^2$ = 0.3218) driven by greater retrieval evidence for retrieve (*M* = 0.0138, *SD* = 0.0392) compared to encode trials (*M* = -0.0161, *SD* = 0.0249). We found a significant main effect of time interval ($F_{19,722}$ = 27.41, $p <$ 0.0001, ${\eta_p}^2$ = 0.419). We did not find a significant main effect of SOA ($F_{3,114}$ = 1.449, $p = 0.2325$, ${\eta_p}^2$ = 0.0367). We found a significant interaction between instruction and time interval ($F_{19,722}$ = 6.479, $p <$ 0.0001, ${\eta_p}^2$ = 0.1457), and a significant interaction between SOA and time interval ($F_{57,2166}$ = 1.345, $p = 0.0448$, ${\eta_p}^2$ = 0.0342). We did not find a significant interaction between instruction and SOA ($F_{3,114}$ = 0.5249, $p = 0.666$, ${\eta_p}^2$ = 0.0136). We did not find a significant three-way interaction between instruction, SOA, and time interval ($F_{57,2166}$ = 0.6392, $p = 0.9838$, ${\eta_p}^2$ = 0.0165). Bayes factor analysis revealed that a model without the three-way interaction term (H_0) is preferred to a model with the three-way interaction term (H_1; H_{10} = 3.164$\times$10ˆ-8 extreme evidence for H_0).

3. We conducted four separate rmANOVAs. Each ANOVA had factors of instruction (encode, retrieve) and time interval (from five to twenty 100 ms time intervals depending on the SOA condition) and retrieval state evidence as the dependent variable. We report the results of this ANOVA in the table below. Bayes factor analysis revealed that a model without instruction (H_0) is preferred to a model with instruction for the four SOA conditions (500 ms SOA: H_1; H_{10} = 0.8890 anecdotal evidence for H_0; 1000 ms SOA: H_1; H_{10} = 0.0377 strong evidence for H_0; 1500 ms SOA: H_1; H_{10} = 0.0001 extreme evidence for H_0; 2000 ms SOA: H_1; H_{10} = 1.157$\times$10ˆ-5 extreme evidence for H_0).

4. We averaged retrieval state evidence across the instruction interval to account for the variable instruction interval length and conducted a 2 $\times$ 4 rmANOVA with factors of instruction (encode, retrieve) and SOA (500 ms, 1000 ms, 1500 ms, 2000 ms) and retrieval state evidence as the dependent variable. We found a significant main effect of SOA ($F_{3,114}$ = 1.729, $p = 0.1650$, ${\eta_p}^2$ = 0.0435). We found greater retrieval evidence for longer SOA compared to shorter SOA conditions. The main effect of instruction and the interaction between instruction and SOA were not significant (main effect: $F_{1,38}$ = 2.595, $p =$ 0.1155, ${\eta_p}^2$ = 0.0639; interaction: $F_{3,114}$ = 1.106, $p = 0.3499$, ${\eta_p}^2$ = 0.0283).

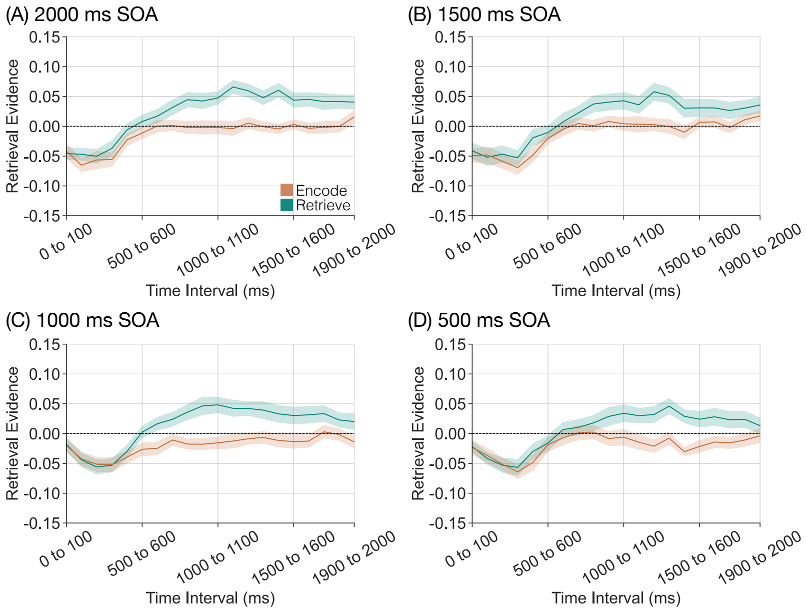

Influence of mnemonic instructions on stimulus-interval retrieval state evidence with N=103 training set. We applied a cross-study classifier to the stimulus interval (twenty 100 ms time intervals) to measure retrieval state evidence as a function of instruction (encode, orange; retrieve, teal) separately for each stimulus onset asynchrony (SOA) condition. **(A-D)** Across all SOA conditions, we find a dissociation in retrieval state evidence on the basis of instruction around 500 ms after stimulus onset. Error bars reflect standard error of the mean.

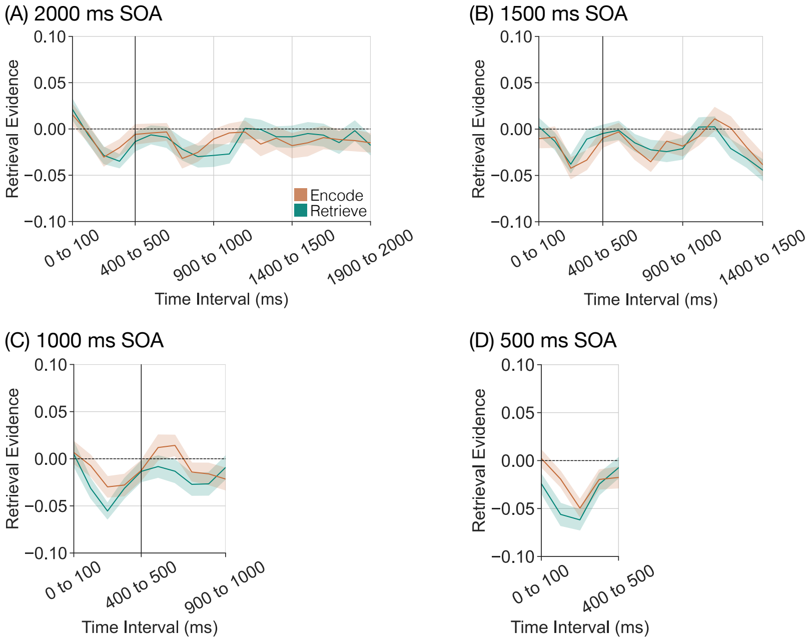

Influence of mnemonic instructions on instruction-interval retrieval state evidence with N=103 training set. We applied a cross-study classifier to the instruction interval (five to twenty 100 ms time intervals depending on the SOA condition) to measure retrieval state evidence as a function of instruction (encode, orange; retrieve, teal) separately for each stimulus onset asynchrony (SOA) condition **(A-D)**. Across all SOA conditions, we find no dissociation in retrieval state evidence on the basis of instruction. The vertical line at the 400 to 500 ms time interval indicates the offset of the instruction cue. Error bars reflect standard error of the mean.

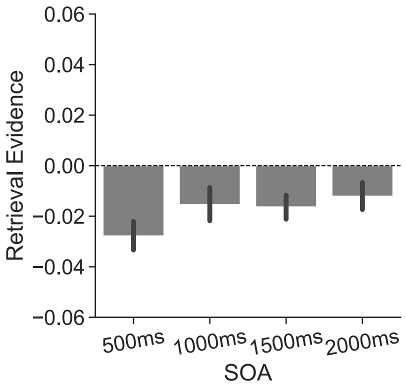

Influence of SOA on instruction-interval retrieval state evidence with N=103 training set. We averaged retrieval state evidence across the instruction interval (five to twenty 100 ms time intervals depending on the SOA condition) and instruction (encode, retrieve) for each SOA condition. We find greater retrieval state evidence for longer SOA conditions. Error bars reflect standard error of the mean.

ANOVA results testing the effect of instruction and time interval on instruction-interval retrieval state evidence separately for each SOA condition with N=103 training set.

	Main effect of inst.	Main effect of time	Interaction of inst. × time
		500 ms	
df	(1,38)	(4,152)	(4,152)
F	2.467	10.88	2.742
p	0.1245	<**0.0001**	0.0307
${\eta_p}^2$	0.061	0.2226	0.0673
		1000 ms	
df	(1,38)	(9,342)	(9,342)
F	2.478	4.935	1.266
p	0.1237	<**0.0001**	0.2544
${\eta_p}^2$	0.0612	0.1149	0.0322
		1500 ms	
df	(1,38)	(14,532)	(14,532)
F	0.0075	4.261	1.008
p	0.9314	<**0.0001**	0.443
${\eta_p}^2$	0.0002	0.1008	0.0259
		2000 ms	
df	(1,38)	(19,722)	(19,722)
F	0.0113	2.48	0.7157
p	0.9159	**0.0005**	0.8049
${\eta_p}^2$	0.0003	0.0613	0.0185

Bold values indicate $p < 0.05$.
